# Correction: Kularbkaew et al. Genetic Variants in the *TBC1D2B* Gene Are Associated with Ramon Syndrome and Hereditary Gingival Fibromatosis. *Int. J. Mol. Sci.* 2024, *25*, 8867

**DOI:** 10.3390/ijms26073228

**Published:** 2025-03-31

**Authors:** Thatphicha Kularbkaew, Tipaporn Thongmak, Phan Sandeth, Teerada Daroontum, Callum S. Durward, Pichai Vittayakittipong, Paul Duke, Anak Iamaroon, Sompid Kintarak, Worrachet Intachai, Chumpol Ngamphiw, Sissades Tongsima, Peeranat Jatooratthawichot, Timothy C. Cox, James R. Ketudat Cairns, Piranit Kantaputra

**Affiliations:** 1 Center of Excellence in Medical Genetics Research, Faculty of Dentistry, Chiang Mai University, Chiang Mai 50200, Thailand; ktonmaii@hotmail.com (T.K.); worrachet.intachai@gmail.com (W.I.); 2 Division of Pediatric Dentistry, Faculty of Dentistry, Chiang Mai University, Chiang Mai 50200, Thailand; 3 Pediatric Division, Hatyai Hospital, Songkhla 90110, Thailand; tipaing@gmail.com; 4 Department of Oral and Maxillofacial Surgery, Preah Ang Duong Hospital, Phnom Penh 120201, Cambodia; phan_sandeth@yahoo.fr; 5 Department of Pathology, Faculty of Medicine, Chiang Mai University, Chiang Mai 50200, Thailand; mewteerada@gmail.com; 6 Faculty of Dentistry, University of Puthisastra, Phnom Penh 120201, Cambodia; callumspencerdurward@gmail.com; 7 Department of Oral and Maxillofacial Surgery, Faculty of Dentistry, Prince of Songkla University, Songkhla 90110, Thailand; pichai.v@hotmail.com; 8 Royal Adelaide Hospital, Adelaide, SA 5000, Australia; paulduke01@gmail.com; 9 Department of Oral Biology and Diagnostic Sciences, Faculty of Dentistry, Chiang Mai University, Chiang Mai 50200, Thailand; iamaroon@yahoo.com; 10 Department of Oral Diagnostic Sciences, Faculty of Dentistry, Prince of Songkla University, Songkhla 90110, Thailand; sompid.k@psu.ac.th; 11 National Biobank of Thailand, National Center for Genetic Engineering and Biotechnology, Thailand Science Park, Pathum Thani 12120, Thailand; chumpol.nga@gmail.com (C.N.); sissades.ton@biotec.or.th (S.T.); 12 School of Chemistry, Institute of Science, and Center for Biomolecular Structure, Function and Application, Suranaree University of Technology, Nakhon Ratchasima 30000, Thailand; pj.cbsfa@gmail.com (P.J.); cairns@sut.ac.th (J.R.K.C.); 13 Departments of Oral & Craniofacial Sciences, School of Dentistry, and Pediatrics, School of Medicine, University of Missouri-Kansas City, Kansas City, MO 64108, USA; coxtc@umkc.edu

## Addition of an Author

The authors wish to make a change to the author names (adding a new author—Teerada Daroontum) on this paper [1].

- The author belongs to the new affiliation 5: ^5^ Department of Pathology, Faculty of Medicine, Chiang Mai University, Chiang Mai 50200, Thailand

Teerada Daroontum was not included as an author in the original publication. The corrected Author Contributions statement appears here.

Dr. Teerada, whose name is missing, worked on immunohistochemical study of the gingiva for us. Dr. Teerada Daroontum has carried out beautiful work on the immunohistochemical study demonstrated in this paper and contributed to the discussion involving it. She has conducted immunohistochemical studies in a number of our published papers. It was my mistake that I [Piranit Kantaputra] failed to add her name as a coauthor.

**Author Contributions:** Conceptualization, T.K., T.T., P.S., T.D., C.S.D., P.V., P.D., A.I., S.K., W.I., C.N., S.T., P.J., T.C.C., J.R.K.C. and P.K.; methodology, T.K., T.T., P.S., T.D., C.S.D., P.V., P.D., A.I., S.K., W.I., C.N., S.T., P.J., T.C.C., J.R.K.C. and P.K.; validation, T.K., T.T., P.S., T.D., C.S.D., P.V., P.D., A.I., S.K., W.I., C.N., S.T., P.J., T.C.C., J.R.K.C. and P.K.; formal analysis, T.K., T.T., P.S., T.D., C.S.D., P.V., P.D., A.I., S.K., W.I., C.N., S.T., P.J., T.C.C., J.R.K.C. and P.K.; investigation, T.K., T.T., P.S., T.D., C.S.D., P.V., P.D., A.I., S.K., W.I., C.N., S.T., P.J., T.C.C., J.R.K.C. and P.K.; resources, P.K.; data curation, T.K., T.T., P.S., T.D., C.S.D., P.V., P.D., A.I., S.K., W.I., C.N., S.T., P.J., T.C.C., J.R.K.C. and P.K.; writing—original draft preparation, T.K., T.T., P.S., T.D., C.S.D., P.V., P.D., A.I., S.K., W.I., C.N., S.T., P.J., T.C.C., J.R.K.C. and P.K.; writing—review and editing, T.K., T.T., P.S., T.D., C.S.D., P.V., P.D., A.I., S.K., W.I., C.N., S.T., P.J., T.C.C., J.R.K.C. and P.K.; supervision, P.K.; project administration, P.K.; funding acquisition, P.K. All authors have read and agreed to the published version of the manuscript.

## Text Correction

There was an error in the original publication [1] in Section 3.3.3.

A correction has been made to Section 3, Section 3.3.3, both in the section header and in paragraph 1.

3.3.3. Genetic Variant in the *KREMEN2* Gene and Dense Mandibular Bone in Patients 3 and 4

In addition to gingival fibromatosis, patients 3 and 4 also had dense mandibular bone. Of note, *KREMEN2* is also critical for bone formation and *Kremen2*-KO mice have been demonstrated to have increased bone formation [41]. Furthermore, overexpression of *Kremen2* in mice resulted in severe osteoporosis with decreased osteoblast and increased osteoclast differentiation and function [41]. Therefore, the genetic variant in the *KREMEN2* gene may also contribute to the dense mandibular bone in patients 3 and 4.

## Figure Correction

There was an error in the original publication [1] in Section 2.2.

A correction has been made to Section 2, Section 2.2, in both Figure 5 and Figure 6.

The two figures were swapped. Figure 5 should be Figure 6, and Figure 6 should be Figure 5. The captions are correct as they currently are.

The authors state that the scientific conclusions are unaffected. This correction was approved by the Academic Editor. The original publication has also been updated.
